# Fluorescent RNA cytosine analogue – an internal probe for detailed structure and dynamics investigations

**DOI:** 10.1038/s41598-017-02453-1

**Published:** 2017-05-24

**Authors:** Anders Foller Füchtbauer, Søren Preus, Karl Börjesson, Scott A. McPhee, David M. J. Lilley, L. Marcus Wilhelmsson

**Affiliations:** 1 Chemistry and Chemical Engineering/Chemistry and Biochemistry, Chalmers University of Technology, Gothenburg, SE-41296 Sweden; 2 Department of Chemistry, University of Copenhagen, Copenhagen, DK-2100 Denmark; 3 Department of Chemistry and Molecular Biology, University of Gothenburg, Gothenburg, SE-41296 Sweden; 4 Cancer Research UK Nucleic Acid Structure Research Group, MSI/WTB Complex, The University of Dundee, Dow Street, Dundee, DD1 5EH UK

## Abstract

The bright fluorescent cytosine analogue tC^O^ stands out among fluorescent bases due to its virtually unquenched fluorescence emission in duplex DNA. However, like most reported base analogues, it has not been thoroughly characterized in RNA. We here report on the first synthesis and RNA-incorporation of tC^O^, and characterize its base-mimicking and fluorescence properties in RNA. As in DNA, we find a high quantum yield inside RNA duplexes (<Φ_F_> = 0.22) that is virtually unaffected by the neighbouring bases (Φ_F_ = 0.20–0.25), resulting in an average brightness of 1900 M^−1^ cm^−1^. The average fluorescence lifetime in RNA duplexes is 4.3 ns and generally two lifetimes are required to fit the exponential decays. Fluorescence properties in ssRNA are defined by a small increase in average quantum yield (<Φ_F _> = 0.24) compared to dsRNA, with a broader distribution (Φ_F_ = 0.17–0.34) and slightly shorter average lifetimes. Using circular dichroism, we find that the tC^O^-modified RNA duplexes form regular A-form helices and in UV-melting experiments the stability of the duplexes is only slightly higher than that of the corresponding natural RNA (<Δ*T*_m_> = + 2.3 °C). These properties make tC^O^ a highly interesting fluorescent RNA base analogue for detailed FRET-based structural measurements, as a bright internal label in microscopy, and for fluorescence anisotropy measurements of RNA dynamics.

## Introduction

In recent years, we have come to understand that the roles of RNA in the cell are many and varied, and go well beyond those of the central dogma to include biocatalysis, transcription and genetic regulation. The discovery two decades ago that short RNA sequences can up- or downregulate gene expression through the RNA interference (RNAi)^1^ pathway sparked high hopes for gene silencing with antisense oligonucleotides (ASOs)^2, 3^, but while RNAi reagents such as siRNA have provided a wealth of information on gene function^4^, their utility in antisense therapy have so far been limited by stability and delivery challenges^2–4^.

The broad range of conformation and functions observed for RNA reflects the importance of the secondary and tertiary structure as well as dynamics, *i.e*. supertertiary structure^5, 6^, for their function, and underline the need for tools that allow a better understanding of these parameters^7, 8^. Traditionally, high-resolution structural insight into nucleic acids has been achieved using nuclear magnetic resonance (NMR) spectroscopy^9^ or X-ray crystallography^10^, often complemented by lower-resolution techniques such as Förster resonance energy transfer (FRET)^11, 12^. Single-molecule FRET opens up new possibilities when it comes to observing biomolecular structure and dynamics in live cells^13, 14^. For in-cell single-molecule FRET measurements, external fluorophores such as Cy-dyes, Alexa-dyes and Atto-dyes have been employed because of their high brightness and photostability^14–17^. However, these probes are less useful for dynamics (anisotropy) and detailed, smaller structural features. If an internal, non-perturbing fluorescent probe is employed, real-time information on the structure and intrinsic dynamics of nucleic acids may also be obtained^12, 18^, which could pave the way towards a deeper understanding of important cellular processes such as the genome editing CRISPR systems^19–23^. It may also support the development of novel oligonucleotide-based therapeutics, such as ASOs, through visualisation of their intercellular transport and subcellular localisation^2^.

The number of internal probes for nucleic acids, especially fluorescent base analogues, has increased considerably over the last decades^24, 25^, and the search for new probes with improved photophysical properties is continuing. However, most of the probes reported to date are not synthesized for or characterized in RNA systems, and the vast majority of these probes are quenched considerably upon incorporation into nucleic acids, the effect being dependent on base sequence, position and whether the nucleic acid is single- or double-stranded^26^. This feature has been extremely useful in studies that probe *e.g*. RNA translation and catalysis^27, 28^ and DNA dynamics^29^. However, for structure and dynamics studies employing FRET or fluorescence anisotropy, or cellular tracking, a bright and stable fluorescent probe would be preferred.

We have previously reported on two internal fluorescent probes based on a tricyclic cytosine scaffold, tC and tC^O^ ^30, 31^. These probes are unique in that they retain their high fluorescence quantum yield (Φ_F_ ~ 0.20) when incorporated into duplex DNA regardless of base sequence or position, positioning them among the brightest internal DNA probes reported to date^31, 32^. We have shown that both probes are excellent cytosine analogues that are rigidly stacked within the duplex, where they have only limited effects on the native conformation of DNA^30, 31^. Both compounds are good substrates for human DNA and RNA polymerases^33, 34^, that have been used to investigate DNA-protein interactions^35, 36^ and to monitor the *i*-motif transition^37, 38^. More recently, we developed the non-emissive FRET acceptor tC_nitro_ that can be used with tC^O^ to accurately distinguish distance- from orientation-changes through internucleobase FRET^39, 40^ and thereby quantitatively resolve solution structures^41^. Even small modifications of the DNA conformation may result in significant changes in the FRET efficiency of these probes, which have been used to gain insight into mammalian mitochondrial transcription^42, 43^, the structure and dynamics of the DNA binding RAD4/XPC complex^44^, and the mechanism and kinetics of the B- to Z-DNA transition^45^.

**Figure 1:**
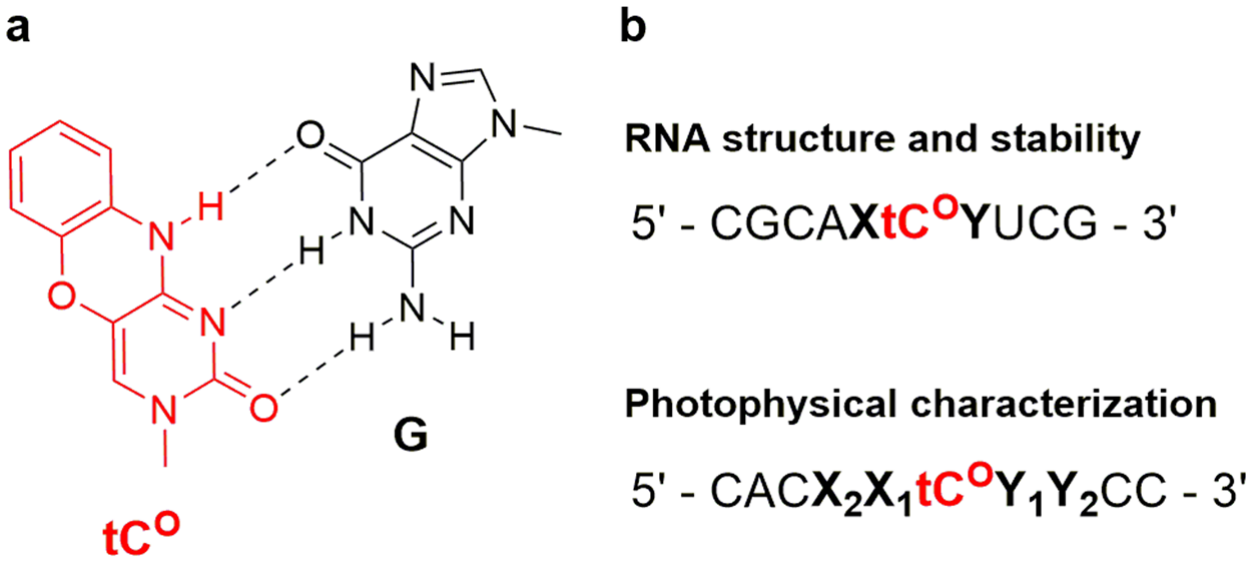
(**a**) The structure of tC^O^ (red) base-paired with guanine (G). (**b**) RNA sequences investigated in this study. Sites of variation in the 5′- and 3′-direction of tC^O^ are denoted with X and Y, respectively. XY and X_2_X_1_Y_1_Y_2_ are given as sequence names in Tables 1 and 2, respectively, and the full sequences can be found in Supplementary Table S1.

Herein, we report the synthesis, RNA-incorporation and characterization of the tC^O^ ribonucleoside, and characterize its base-mimicking and fluorescence properties as a probe for RNA systems. Two series of 10-mer RNA-sequences were studied (Fig. 1): First, the effects of tC^O^-incorporation into four RNA duplexes were investigated by circular dichroism and UV-melting experiments to ensure that tC^O^ does not significantly perturb the RNA structure. A second series of nine decamer sequences were used to investigate the photophysical properties of tC^O^ in single- and double-stranded RNA (ssRNA and dsRNA) in detail. Importantly, tC^O^ has a high fluorescence quantum yield inside RNA duplexes that is virtually unaffected by the nature of the surrounding base pairs. These unique properties should make tC^O^ a useful internal RNA probe for detailed FRET structure measurements, in fluorescence microscopy, and for probing the dynamics of subdomains of complex RNA 3D-structures with fluorescence anisotropy.

## Results and Discussion

### Synthesis of the tC^O^ phosphoramidite monomer for incorporation into RNA oligonucleotides

**Figure 2:**
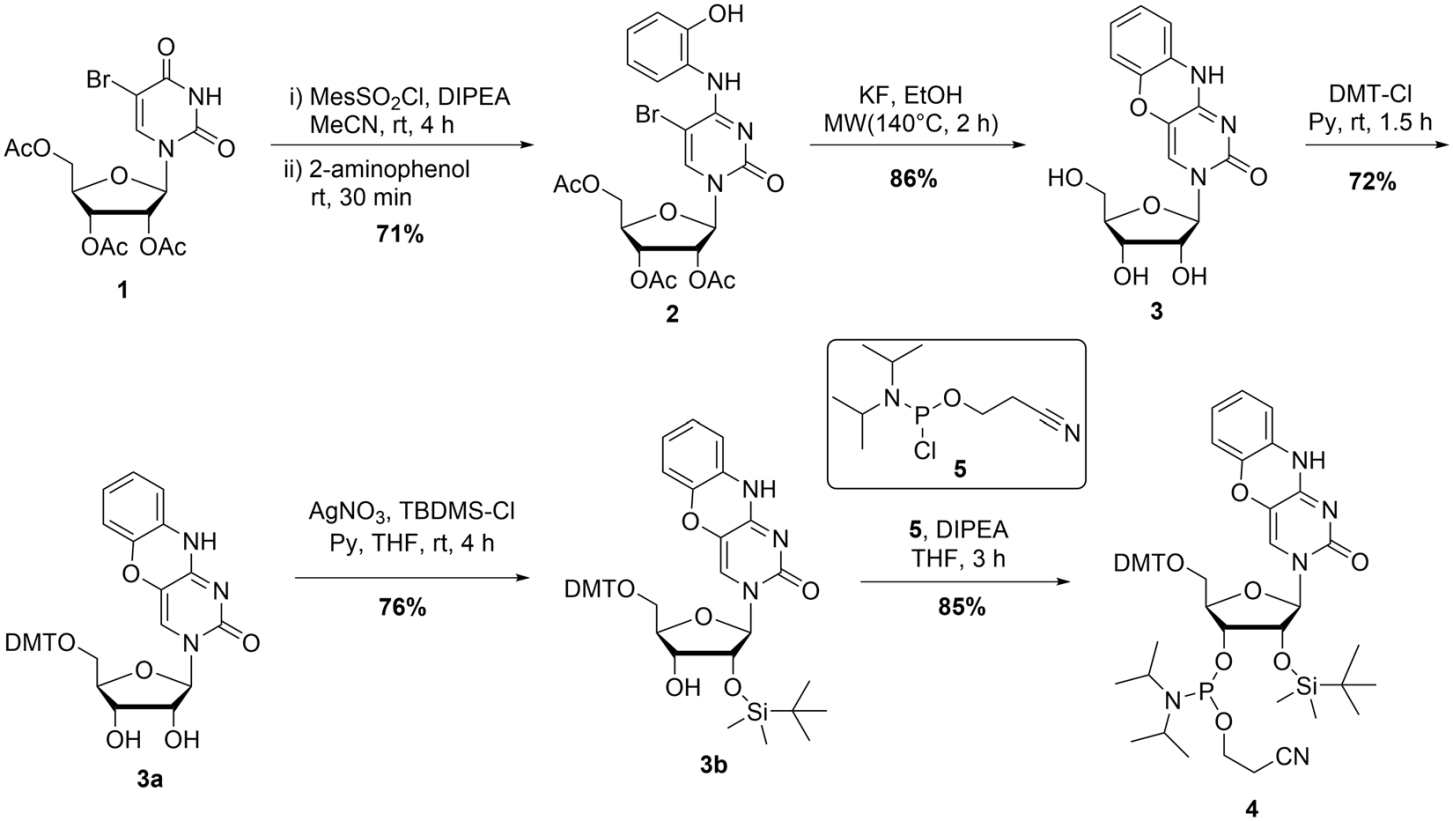
Synthesis of the tC^O^ ribonucleoside phosphoramidite monomer.

The tC^O^-ribonucleoside was synthesized for the first time in a two-step protocol, followed by three protection steps, to afford the tC^O^ phosphoramidite monomer (**4**) (Fig. 2). This approach was influenced by the synthesis of the deoxy-tC^O^ nucleoside by Lin and co-workers^46^. In short, activation of the O-4 of 2′,3′,5′-triacetyl-5-bromouridine (**1**) with 2-mesitylenesulfonyl chloride produced an active O-4-sulphonate ester, which was reacted with 2-aminophenol to produce **2**. Deprotection and cyclization was achieved in one pot, using KF to facilitate the cyclization, to produce **3**. Finally, the tC° ribonucleoside was protected with 5′-*O*-(4,4’-dimethoxytrityl) (DMT), followed by 2′-*O*-*tert*-butyldimethylsilyl (TBDMS) and a 3′-phosphoramidite for incorporation into RNA oligonucleotides using solid-phase synthesis (see Supplementary Table S1).

### Structure and stability of tC^O^-modified RNA oligonucleotides

In a preliminary study, a small-scale batch of four modified 10-mer RNA sequences was synthesized, differing only in the bases neighbouring tC^O^ (Fig. 1B and Table 1). To investigate the effect of tC^O^-incorporation on the structure and stability of RNA duplexes, circular dichroism (CD) as well as melting temperature was measured on both the modified RNA duplexes and the corresponding reference duplexes.

**Table 1:** Melting temperatures of tC^O^-containing RNA duplexes (*T*_m_tC^O^), the corresponding unmodified duplexes (*T*_m_^UM^) and the difference in melting temperature as a result of changing one C for tC^O^ (Δ*T*_m_).

Sequence^a^	Sequence name	*T*_m_tC^O^ [°C]^b^	*T*_m_^UM^ [°C]^b^	Δ*T*_m_ [°C]
5′-CGCA**UtC** ^**o**^ **A**UCG-3′	**UA**	56	55	+1
5′-CGCA**UtC** ^**o**^ **U**UCG-3′	**UU**	57	56	+1
5′-CGCA**CtC** ^**o**^ **U**UCG-3′	**CU**	68	63	+5
5′-CGCA**GtC** ^**o**^ **G**UCG-3′	**GG**	69	67	+2

^a^In unmodified sequences, tC^O^ is replaced by cytosine. ^b^Measurements were performed in PBS buffer (100 mM Na^+^, pH 7.5). *T*_m_ values were determined as the average of the temperature values at the maximum of the first derivative and at half maximum of the melting curves.

Circular dichroism spectra (see Supplementary Fig. S1) of all four tC^O^-modified sequences exhibit the general characteristics of A-form RNA, namely a positive band at 265 nm and a strong negative band at 210 nm, indicating that when a cytosine is replaced with tC^O^ in a RNA duplex, the RNA adopts A-form geometry. In a similar manner to tC^O^ in DNA, no significant CD signal was found for the long wavelength absorption band of tC^O^ ^31^. The thermal stability of the modified and the corresponding unmodified RNA duplexes is summarized in Table 1. On average, incorporation of tC^O^ increases the melting temperature by 2.3 °C. A similar increase in stability (2.7 °C) was observed for tC- and tC^O^-incorporation into DNA, and presumably reflects that the increased base stacking interactions act to stabilize the duplex structure^30, 31^. In the few reports on fluorescent base analogues in RNA, a slightly stabilizing effect was also reported for the emissive guanosine isomorph, ^th^G^47^, whereas a negligible effect was reported for the uridine analogue U^Dz^ ^48^, and a destabilizing effect was reported for benzo*[b]*thiophene-^49^, benzofuran-^50^ and naphthalimide-conjugated^51^ uridine analogues.

### Photophysical properties of tC^O^ in single-stranded RNA

Having established that RNA duplexes retains their native A-helix conformation and are slightly stabilized after incorporating tC^O^, we prepared nine new decamer sequences from a larger batch of tC^O^ ribonucleoside for investigation of the photophysical properties of tC^O^ in single- and double-stranded RNA (ssRNA and dsRNA). Three of the sequences (GUUU, GAUU and UUUG) were chosen to investigate possible effects from guanines positioned two bases away from tC^O^, as a slight quenching effect of tC^O^ by guanines have been observed in DNA^31^.

**Figure 3:**
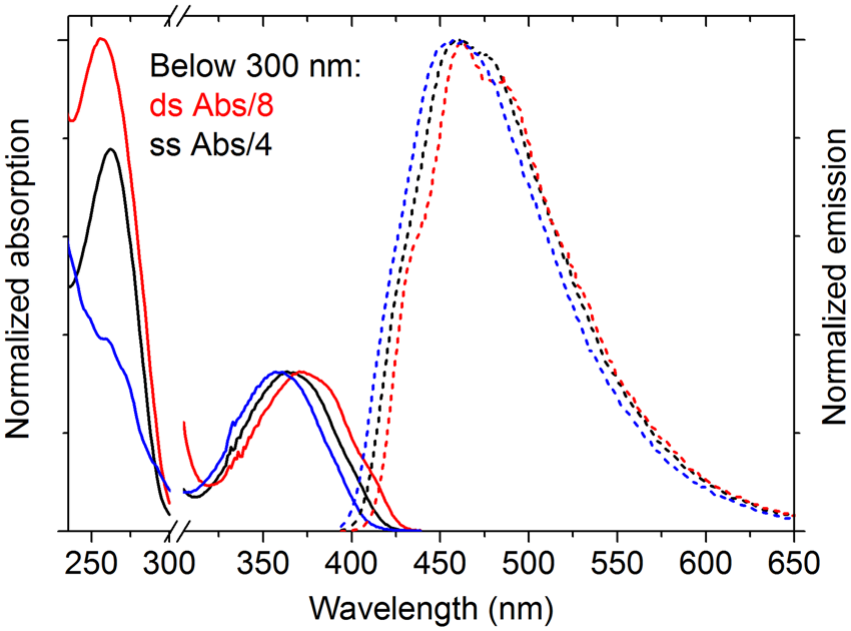
Isotropic absorption (solid) and emission (dashed) spectra of the tC^O^-monomer (blue), tC^O^-containing RNA single strand (AU, black) and tC^O^-containing RNA duplex (AU, red). For better comparison of spectral envelopes, the spectra have been normalized at the maxima of the lowest energy absorption band (left) and of the emission (right), respectively.

Figure 3 shows representative absorption and emission spectra of tC^O^ in single- and double-stranded RNA together with the corresponding spectra of tC^O^ as a monomer. As is the case for DNA, the long-wavelength absorption maximum is slightly red-shifted upon incorporation in ssRNA (362–368 nm) compared to the tC^O^ monomer (359 nm)^31^. The emission spectrum of tC^O^ in ssRNA shows slightly more vibrational fine structure than that of the monomer, with an emission maximum (453–458 nm) very close to that of the monomer (457 nm). Similar spectral effects were observed for tC^O^ in DNA and is common for fluorescent base analogues incorporated into nucleic acids^24, 25^.

Table 2 lists the fluorescence quantum yield, Φ_F_, mean fluorescence lifetimes, <τ>, and calculated radiative rate constants, k_r_, and non-radiative rate constants, k_nr_, of tC^O^ in ssRNA. The fluorescence decays could all be fitted with two fluorescence lifetimes (see Supplementary Fig. S2). When comparing the mean fluorescence lifetimes of all sequences, the six sequences where tC^O^ is flanked by a uracil have a shorter lifetime (2.6–3.3 ns) than both the free monomer (3.4 ns) and the other three single-strands, GG, AA and CC (4.7–5.4 ns). These six sequences also have a reduced fluorescence quantum yield and a higher non-radiative rate constant (Φ_F_ = 0.17–0.22, k_nr_ = 2.4–3.2·10^8^ s^−1^) compared to the free monomer (Φ_F_ = 0.30, k_nr_ = 2.1·10^8^ s^−1^) as well as the other three sequences (Φ_F_ = 0.31–0.34, k_nr_ = 1.2–1.5·10^8^ s^−1^). Interestingly, this quenching effect appears to be similar regardless of whether the uracil is positioned on the 3′ side or the 5′ side (*cf*. sequences UG and GU). The effect is not increased significantly in the two sequences where tC^O^ is positioned between two uracils (GUUU and UUUG), and the differences observed in quantum yield and mean fluorescence lifetime between them is too small for us to conclude whether the quenching is dependent on the relative orientation and interaction between tC^O^ and its flanking uracils. Comparing sequences AU with GAUU and GUUU with UUUG, we find that replacing a uracil with guanine two steps away from tC^O^ only has a small or negligible effect on the fluorescence quantum yield and average fluorescence lifetime. In ssDNA, sequences containing a guanine on the 5′ side to tC^O^ were shown to have shorter lifetimes (2.8–3.0 ns) and quantum yield (Φ_F_ = 0.14–0.17) compared to other sequences (<τ> = 4.4–5.8 ns and Φ_F_ = 0.29–0.41), whereas sequences with guanine on the 3′ side were among the brightest, suggesting that subtle structural differences such as relative orientation and stacking plays an important role^31^.

**Table 2:** Optical properties of the tC^O^ monomer and tC^O^-containing RNA single strands.

Sequence name^a^	λ_Abs,max_ [nm]^b^	λ_Em,max_ [nm]^b^	Φ_F_^b,c^	τ_1_(α_1_) [ns]^b,d^	τ_2_(α_2_) [ns]^b,d^	<τ>[ns]^e^	k_r_ [10^7^ s^−1^]^f^	k_nr_ [10^8^ s^−1^]^g^
**tC** ^**O**^ **monomer**	359	457	0.30	3.4 (1.00)	—	3.4	8.8	2.1
**GG**	367	455	0.31	5.7 (0.71)	2.4 (0.29)	4.7	6.6	1.5
**UG**	364	456	0.21	4.1 (0.63)	1.6 (0.37)	3.2	6.6	2.5
**GU**	364	458	0.19	3.9 (0.57)	1.4 (0.43)	2.8	6.8	2.9
**AA**	368	458	0.34	5.8 (0.89)	2.0 (0.11)	5.4	6.3	1.2
**AU**	363	457	0.22	4.3 (0.64)	1.4 (0.36)	3.3	6.7	2.4
**CC**	368	453	0.33	5.6 (0.80)	2.2 (0.20)	4.9	6.7	1.4
**GUUU**	364	457	0.17	3.9 (0.50)	1.3 (0.50)	2.6	6.5	3.2
**GAUU**	364	457	0.22	4.4 (0.62)	1.5 (0.38)	3.3	6.7	2.4
**UUUG**	362	458	0.21	4.1 (0.63)	1.4 (0.37)	3.1	6.8	2.5

^a^Sequences are named after the bases flanking tC^O^ (see Fig. 1b). Full sequences can be found as Supplementary Table S1. ^b^Measurements were performed in PBS buffer (100 mM Na^+^, pH 7.5). ^c^Fluorescence quantum yields are measured relative to the quantum yield of the potassium salt of the tC^O^-monomer in water (Φ_F_ = 0.30)^31^. ^d^The amplitudes are indicated in parenthesis. ^e^Mean fluorescence lifetime <τ> = ∑α_i_τ_i_/∑α_i_. ^f^Radiative rate constant, k_r_ = Φ_F_/<τ>. ^g^Non-radiative rate constant, k_nr_ = k_r_/Φ_F_ − k_r_.

### Structural and photophysical properties of tC^O^ in double-stranded RNA

Representative absorbance and emission spectra of tC^O^ in double-stranded RNA is included in Fig. 3. The long-wavelength absorption maximum is slightly red-shifted (368–373 nm) compared to that of single strands (362–368 nm), and there is a very weak vibrational fine structure on the long wavelength side of the absorption spectra. Although the overall emission peak is slightly red-shifted, the vibrational fine structure observed in the emission spectra results in an emission maximum (452–460 nm) that is not shifted significantly compared to the monomer (457 nm). The vibrational fine structure in both the absorbance and emission spectra of tC^O^ in dsRNA was also observed for tC^O^ in dsDNA, indicating that tC^O^ is firmly stacked inside the RNA helix as has previously been found in DNA^31^.

**Figure 4:**
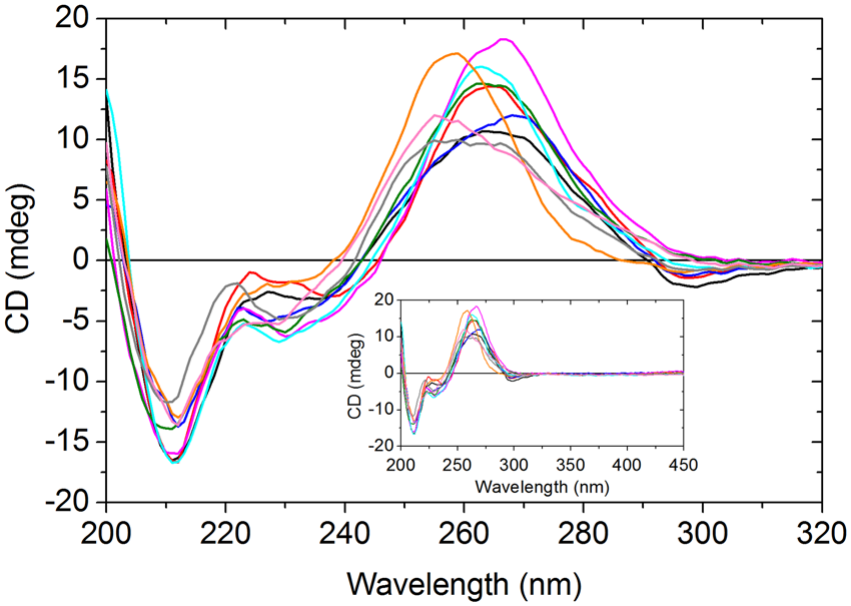
Circular dichroism spectra of nine RNA duplexes containing tC^O^. Duplexes are denoted by the bases neighbouring tC^O^ according to Fig. 1b, and consist of the modified strands GG (black), UG (red), GU (blue), AA (orange), AU (pink), CC (gray), GUUU (purple), GAUU (green) and UUUG (cyan) hybridized to the complementary natural RNA strand. In the insert, the wavelength range is extended to 450 nm.

CD spectra (Fig. 4) on all nine tC^O^-modified RNA duplexes show the general characteristics of A-form RNA, namely a positive band at 265 nm and a strong negative band at 210 nm. The long-wavelength band of tC^O^ is not observed in any of the CD spectra (see insert Fig. 4). For other base analogues such as the parent compound tC and 2-AP, the long wavelength absorption band can indeed be observed in CD. So far no satisfactory explanation for this has been found, but the same behaviour was observed in the CD spectra of tC^O^ in dsDNA^31^.

Table 3 lists the photophysical properties of tC^O^ when incorporated in double-stranded RNA. Overall, there is less variation between the fluorescence quantum yields and the mean fluorescence lifetimes of the double-stranded sequences compared to the single-stranded ones. The fluorescence quantum yield in dsRNA is slightly decreased compared to the free monomer and is relatively insensitive to the neighbouring bases (Φ_F_ = 0.20–0.25). A similar trend has been observed for tC^O^ in dsDNA (Φ_F_ = 0.22 ± 0.05)^31^. Inside dsDNA, tC and tC^O^ are one of the few internal base analogues that have a single lifetime (<τ > = 3.4–4.8 ns for tC^O^), which is a great advantage in techniques such as fluorescence anisotropy and FRET. However, while the fluorescence decays of tC^O^ in dsRNA can be fitted using a single lifetime, slightly better fits are obtained when using two lifetimes: a large component of 4.1–4.9 ns and a smaller component of ~2 ns, resulting in mean fluorescence lifetimes ranging between 3.8 and 4.7 ns (see Supplementary Fig. S3). The calculated radiative and non-radiative rate constants for the tC^O^-containing double strands range between 4.9·10^7^ and 5.6·10^7^ s^−1^, and between 1.7·10^8^ and 2.1·10^8^ s^−1^, respectively. These values are comparable with the values for tC^O^ in dsDNA (k_r_ = 4.6–5.6·10^7^ s^−1^; k_nr_ = 1.5–2.3·10^8^ s^−1^). Much like in dsDNA, the radiative rate constant is reduced by ~40% compared to the tC^O^ monomer (k_r_ = 8.8·10^7^ s^−1^), most likely a consequence of the electronic interaction between tC^O^ and other bases in the DNA, leading to a hypochromic effect^31^. This would result in a reduction of the radiative rate constant, since it is proportional to the extinction coefficient according to the Strickler-Berg relation^52^. The observed change in the radiative rate constant is similar to the hypochromicity of dsDNA as compared to a free nucleoside, which is typically <40%^53^. Altogether, our investigations of the structural and photophysical properties in double-stranded RNA suggest that tC^O^ is a very promising non-perturbing internal RNA fluorophore.

**Table 3:** Optical properties of tC^O^-containing RNA double strands.

Sequence^a^	λ_Abs,max_ [nm]^b^	λ_Em,max_ [nm]^b^	Φ_F_^b,c^	τ_1_(α_1_) [ns]^b,d^	τ_2_(α_2_) [ns]^b,d^	<τ>[ns]^e^	k_r_ [10^7^ s^−1^]^f^	k_nr_ [10^8^ s^−1^]^g^
**GG**	373	453	0.22	4.9 (0.87)	1.9 (0.13)	4.5	4.9	1.7
**UG**	368	452	0.20	4.1 (0.88)	2.1 (0.12)	3.8	5.3	2.1
**GU**	373	460	0.22	4.8 (0.88)	2.2 (0.12)	4.5	4.9	1.7
**AA**	371	457	0.23	4.6 (0.89)	2.1 (0.11)	4.3	5.3	1.8
**AU**	370	459	0.23	4.4 (0.90)	2.0 (0.10)	4.2	5.5	1.8
**CC**	371	452	0.25	4.9 (0.91)	2.1 (0.09)	4.7	5.3	1.6
**GUUU**	370	456	0.22	4.5 (0.87)	2.2 (0.13)	4.2	5.2	1.9
**GAUU**	373	459	0.22	4.6 (0.88)	2.2 (0.12)	4.3	5.1	1.8
**UUUG**	371	456	0.23	4.3 (0.89)	1.9 (0.11)	4.1	5.6	1.9

^a^Sequences are named after the bases flanking tC^O^ (see Fig. 1b). Full sequences can be found in Supplementary Table S1. ^b^Measurements were performed in PBS buffer (100 mM Na^+^, pH 7.5). ^c^Fluorescence quantum yields are measured relative to the quantum yield of the potassium salt of the tC^O^-monomer in water (Φ_F_ = 0.30)^31^. ^d^The amplitudes are indicated in parenthesis. ^e^Mean fluorescence lifetimes < τ >  = ∑α_i_τ_i_/∑α_i_. ^f^Radiative rate constant, k_r_ = Φ_F_/<τ>. ^g^Non-radiative rate constant, k_nr_ = k_r_/Φ_F_ − k_r_.

## Conclusion

We have, for the first time, shown that the base analogue tC^O^ can be incorporated into RNA, where it preserves the A-form duplex and slightly stabilizes it. The tC^O^ ribonucleoside can be synthesized through a simple and straight-forward synthetic protocol that affords the phosphoramidite-protected tC^O^ ribonucleoside in high yield. As is the case for DNA, tC^O^ retains its high fluorescence quantum yield inside RNA duplexes, and is virtually unaffected by the nature of the surrounding base pairs. Because of these properties, the tC^O^-ribonucleoside is potentially a valuable fluorescent RNA base analogue whose average brightness (ε·Φ_F_) of 1900 M^−1^ cm^−1^ in dsRNA is the greatest for any internal RNA analogue reported to date. As we have previously demonstrated for DNA-systems, we envisage its main advantages in RNA to be in detailed FRET-based structural measurements, where there is a current lack of internal FRET-probes, as a bright label in microscopy, and in fluorescence anisotropy probing the dynamics of subdomains of complex RNA 3D-structures.

## Methods

### Materials and instruments

Commercially available reagents were used without further purification. Deoxygenation of reaction mixtures was achieved by bubbling argon through the solution for 30 min. Column chromatography were performed using silica gel (Matrex, LC 60 Å/35–70 μm). ^1^H (400 MHz) and ^13^C (100.6 MHz) NMR spectra were recorded at room temperature using a Jeol Eclipse 400 NMR spectrometer. All shifts are recorded in ppm relative to the deuterated solvent (CDCl_3_, THF-*d*_8_ or DMSO-*d*_6_). Positive FAB high resolution mass spectra were obtained on a JEOL SX102 mass spectrometer at Instrumentstationen, Lund University, Sweden. Samples were desorbed from a 3-NBA matrix using 6 kV xenon atoms. Synthesis and characterization data of the synthesized compounds can be found in the Supplementary Information online.

### Incorporation of tC^O^ in RNA-oligonucleotides and their purification

Oligoribonucleotides were synthesized using UltraMILD ribonucleotide phosphoramidites (Link Technologies) with 2′-*O*-*tert*-butyldimethylsilyl (TBDMS) protection implemented on an Applied Biosystems 394 synthesizer^1^. Oligoribonucleotides were cleaved from the support and base deprotected in 25% ethanol/ammonia solution at 20 °C for 3 h, and evaporated to dryness. Removal of TBDMS protecting groups was achieved by redissolving oligoribonucleotides in 115 μL dimethyl sulfoxide to which was added 125 μL 1 M triethylamine trihydrofluoride (Sigma-Aldrich) and incubated at 65 °C for 2.5 h prior to butanol precipitation. All oligonucleotides were purified by gel electrophoresis in 20% polyacrylamide under denaturing conditions (7 M urea) in 90 mM Tris-borate (pH 8.3), 10 mM EDTA (TBE buffer). The full-length RNA product was visualized by brief ultraviolet shadowing. The band was excised and electroeluted using an Elutrap (Whatman) into 45 mM Tris-borate (pH 8.5), 5 mM EDTA buffer, 8 M ammonium chloride at 200 V. The RNA was precipitated with ethanol, washed with 70% ethanol, dried and resuspended in water. Oligoribonucleotides were subjected to further purification by reversed-phase HPLC (ACE C18-AR, Advanced Chromatography Technologies), using an acetonitrile gradient with an aqueous phase of 100 mM triethylammonium acetate (pH 7.0).

### Oligonucleotide preparation

All samples used in this study, unless stated otherwise, were prepared in a sodium phosphate buffer, pH 7.5 with 100 mM added NaCl and 1 mM EDTA. All samples were mixed and handled in sterile, RNase-free environments. The oligonucleotide concentration was determined by measuring the absorption at 260 nm. The molar absorptivities of the unmodified oligonucleotide single strands at 260 nm were calculated in IDT’s online oligonucleotide analyzer^54^. Molar absorptivities of modified strands were calculated in the same way, with the modified base replaced by cytosine, and correcting for the molar absorptivity difference between tC^O^ (ε_tC_^O^ = 11000 M^−1^ cm^−1^) and cytosine (ε_C_ = 7400 M^−1^cm^−1^) at 260 nm. The total extinction coefficient of each sequence can be found in Supplementary Table S1. Double-stranded oligonucleotides were formed by mixing equimolar amounts of complementary single strands in phosphate buffer at room temperature. In fluorescence measurements, an excess of 20% of the non-fluorescent complementary strand was used to ensure that no single-stranded modified sequences were present after hybridization. Hybridization was performed by heating the samples to 85 °C followed by cooling to 5 °C at a rate of 1 °C/min.

### UV-detected thermal melting

Melting curves were recorded on a Cary 4000 spectrophotometer (Varian Technologies) with a programmable multi-cell temperature block, by heating the samples to 85 °C at 0.5 °C/min, followed by cooling to 5 °C at a rate of 0.5 °C/min. The temperature was kept at 85 °C for 5 min between heating and cooling. For unmodified duplexes a temperature range of 15 °C to 92 °C was used. The absorption at 260 nm was recorded every 0.5 °C. Melting temperatures presented in this article are averages of the temperature values at the maximum of the first derivative and at half maximum of the melting curves, and were measured at least twice.

### Circular dichroism

Circular dichroism (CD) spectra were recorded on a Chirascan CD spectrometer (Applied Photophysics) at 25 °C. Spectra of solutions containing 7 mM RNA duplexes, prepared as described above, were recorded between 200 and 450 nm at a scan rate of 1 nm/s. All spectra were corrected for background contributions, and smoothed (3-point adjacent-averaging).

### Steady-state fluorescence

Quantum yields (Φ_F_) of the different tC^O^-modified RNA oligonucleotides were determined relative to the quantum yield of the potassium salt of a tC^O^-monomer in H_2_O (Φ_F_ = 0.30)^31^. Samples containing duplex RNA were prepared as described above. The single-stranded RNA oligonucleotides were set to have an absorption around 0.05 at the excitation wavelength. Spectra were recorded on a SPEX fluorolog 3 spectrofluorimeter (JY Horiba). The samples were excited at 358 nm, and emission spectra were recorded between 385 and 710 nm.

### Time-resolved fluorescence

Fluorescence lifetimes were measured using time-correlated single-photon counting (TCSPC). The excitation pulse was generated by a PicoQuant pulsed (10 MHz) laser diode, emitting at 377 nm. The emission was monitored at 470 nm. Photons were collected by a microchannel-plate photomultiplier tube (MCP-PMT R3809U-50; Hamamatsu) and fed into a multichannel analyzer (Edinburgh Analytical Instruments) with 4096 channels. 10000 counts were recorded in the top channel. The intensity data were convoluted with the instrument response function and fitted to mono-, bi- or tri-exponential expressions using Fluofit Pro v.4 software (PicoQuant GmbH). The average lifetimes were amplitude-weighed: <τ> = ∑α_i_τ_i_/∑α_i_, where <τ> is the average lifetime, τ_i_ is the i^th^ lifetime and α_i_ is the amplitude of the i^th^ lifetime.

## Electronic supplementary material

Supplementary Information
