# Supplementary material for: Fluorescent RNA cytosine analogue – an internal probe for detailed structure and dynamics investigations
Source: Sci Rep. 2017 May 24;7:2393. doi: 10.1038/s41598-017-02453-1 (PMC5443824; doi:10.1038/s41598-017-02453-1)
Supplement: Supplementary file 1 — Supplementary Information [file 41598_2017_2453_MOESM1_ESM.doc]

**Fluorescent RNA cytosine analogue – an internal probe for detailed structure and dynamics investigations**

Anders Foller Füchtbauer^1^, Søren Preus^2^, Karl Börjesson^3^, Scott A. McPhee^4^, David M.J. Lilley^4^, and L. Marcus Wilhelmsson^1^,*

^1^ Chemistry and Chemical Engineering/ Chemistry and Biochemistry, Chalmers University of Technology, Gothenburg, SE-41296, Sweden

^2^ Department of Chemistry, University of Copenhagen, Copenhagen, DK-2100, Denmark

^3^ Department of Chemistry and Molecular Biology, University of Gothenburg, Gothenburg, SE-41296, Sweden

^4^ Cancer Research UK Nucleic Acid Structure Research Group, MSI/WTB Complex, The University of Dundee, Dow Street, Dundee DD1 5EH, UK

# * To whom correspondence should be addressed. Tel: +46 31 7723051; Fax: +46 31 7723858; Email: marcus.wilhelmsson@chalmers.se

# ****Supplementary material****

Supplementary Table S1. Total extinction coefficients of tC^O^-modified and unmodified RNA sequences

Supplementary Figure S1. CD spectra of the RNA duplexes shown in Table 1.

Supplementary Figure S2. Fitted fluorescence decays of tC^O^ in single stranded RNA.

Supplementary Figure S3. Fitted fluorescence decays of tC^O^ in double stranded RNA.

Synthesis and characterization data

**Spectral Data**

References

****Supplementary Table S1. Total extinction coefficients of tC^O^-modified and unmodified RNA sequences****

| Sequence^a^ | Sequence name | ε_260_(tC^O^-modified) [M^-1^·cm^-1^]^a^ | ε_260_(unmodified) [M^-1^·cm^-1^]^a^ | ε_260_(complementary strand) [M^-1^·cm^-1^]^a,b^ |
| --- | --- | --- | --- | --- |
| 5’-CGCA**UtC^O^A**UCG-3’ | **UA** | 97300 | 93700 | 99100 |
| 5’-CGCA**UtC^O^U**UCG-3’ | **UU** | 93600 | 90000 | 102000 |
| 5’-CGCA**CtC^O^U**UCG-3’ | **CU** | 90300 | 86700 | 99500 |
| 5’-CGCA**GtC^O^G**UCG-3’ | **GG** | 94800 | 91200 | 89200 |
| 5’-CACU**GtC^O^G**UCC-3’ | **GG** | 89800 | 86200 | 100600 |
| 5’-CACU**UtC^O^G**UCC-3’ | **UG** | 90800 | 87200 | 106600 |
| 5’-CACU**GtC^O^U**UCC-3’ | **GU** | 88200 | 84600 | 106000 |
| 5’-CACU**AtC^O^A**UCC-3’ | **AA** | 96200 | 92600 | 107800 |
| 5’-CACU**AtC^O^U**UCC-3’ | **AU** | 92500 | 88900 | 110700 |
| 5’-CACU**CtC^O^C**UCC-3’ | **CC** | 83800 | 80200 | 108200 |
| 5’-CAC**GUtC^O^UU**CC-3’ | **GUUU** | 90800 | 87200 | 106600 |
| 5’-CAC**GAtC^O^UU**CC-3’ | **GAUU** | 93100 | 89500 | 104300 |
| 5’-CAC**UUtC^O^UG**CC-3’ | **UUUG** | 88200 | 84600 | 106000 |

**^a^ Calculated in IDT’s online oligonucleotide analyzer (**[**http://eu.idtdna.com/calc/analyzer**](http://eu.idtdna.com/calc/analyzer)**). ^b^ Calculated for the unmodified RNA-sequence that is complementary to the one shown.**

#

Supplementary Figure S1. CD spectra of the RNA duplexes shown in Table 1. A-form RNA is characterized by a dominant positive band at 265 nm, a strong negative band at 210 nm and a negative band at 290 nm.


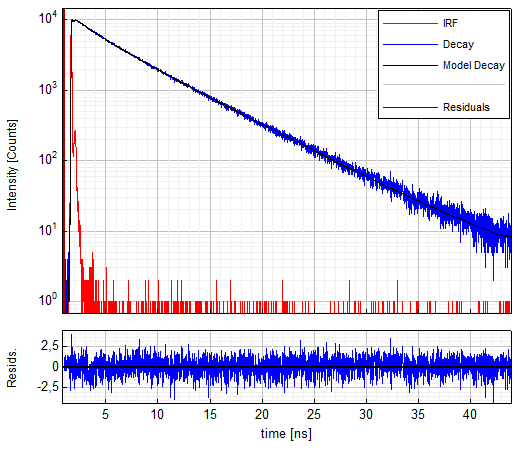

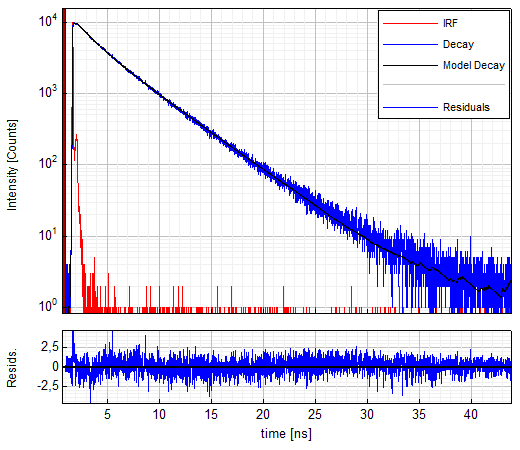


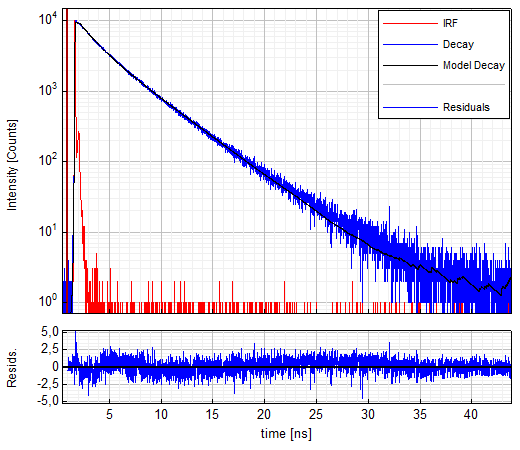

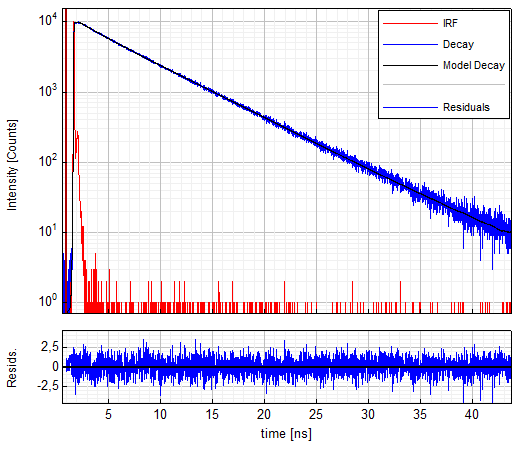


**ssUG**

**ατ**0.6274.110.3731.64

χ^2^(red) = 1.012

**ssGG**

**ατ**0.7115.700.2892.38

χ^2^(red) = 1.017

**ssGU**

**ατ**0.5673.940.4331.41

χ^2^(red) = 1.116

**ssAA**

**ατ**0.8885.840.1122.04

χ^2^(red) = 1.025

**ssAU**

**ατ**0.6404.340.3601.42

χ^2^(red) = 1.034

**ssCC**

**ατ**0.7995.610.2012.24

χ^2^(red) = 1.004


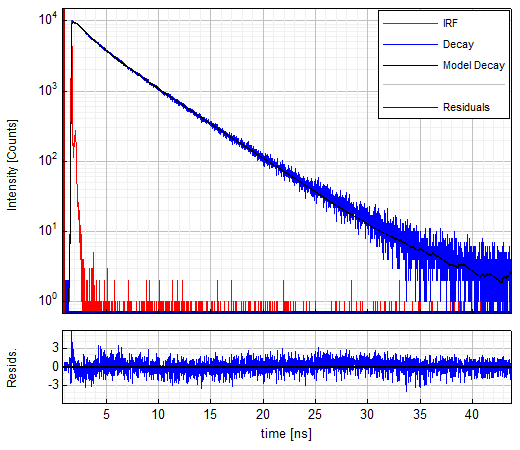

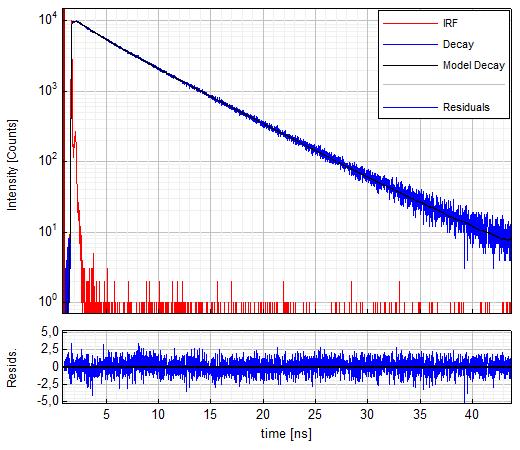


**Supplementary Figure S2. Fitted fluorescence decays of tC^O^ in single stranded RNA.**


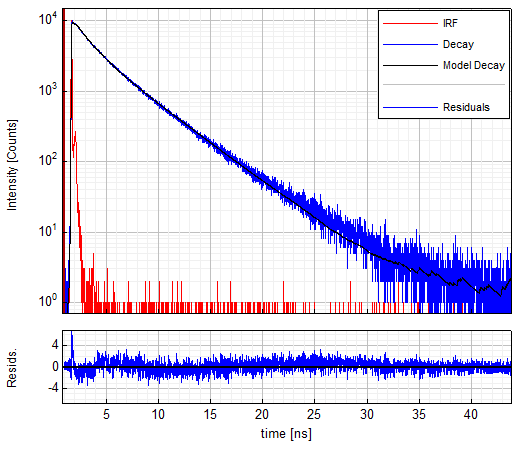

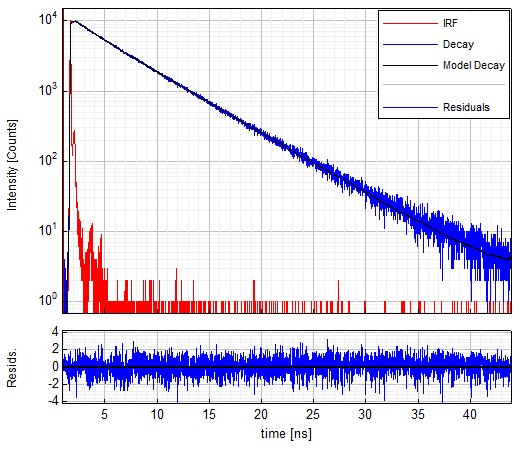


**ssGUUU**

**ατ**0.4983.880.5021.34

χ^2^(red) = 1.088


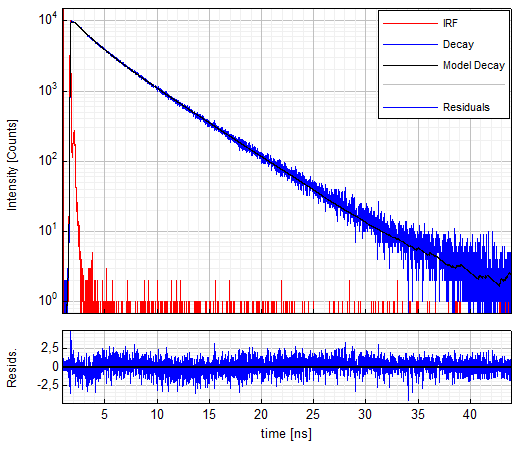

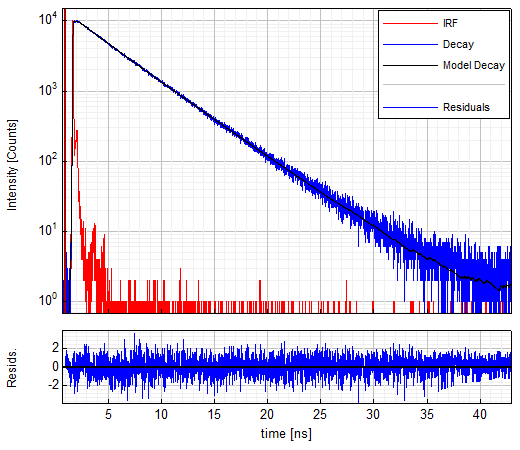


**dsGG**

**ατ**0.8734.940.1271.90

χ^2^(red) = 0.987

**ssGAUU**

**ατ**0.6234.420.3771.54

χ^2^(red) = 1.082

**ssUUUG**

**ατ**0.6334.140.3671.44

χ^2^(red) = 0.991

**dsGU**

**ατ**0.8844.770.1162.19

χ^2^(red) = 0.964

**dsUG**

**ατ**0.8824.060.1182.12

χ^2^(red) = 0.946


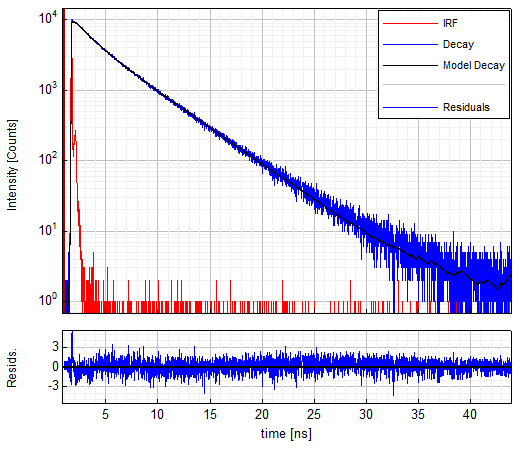

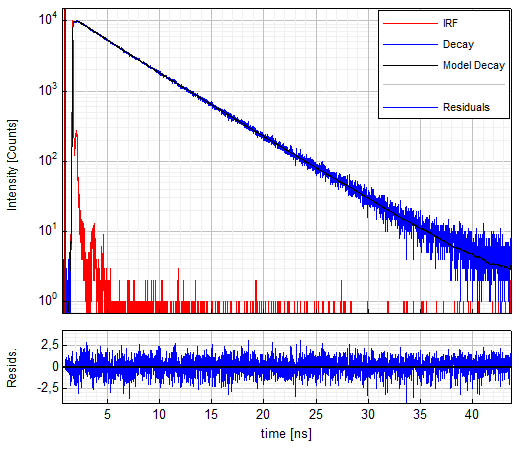


**Supplementary Figure S2. Fitted fluorescence Supplementary Figure S3. Fitted fluorescence
decays of tC^O^ in single stranded RNA (cont.). decays of tC^O^ in double stranded RNA.**


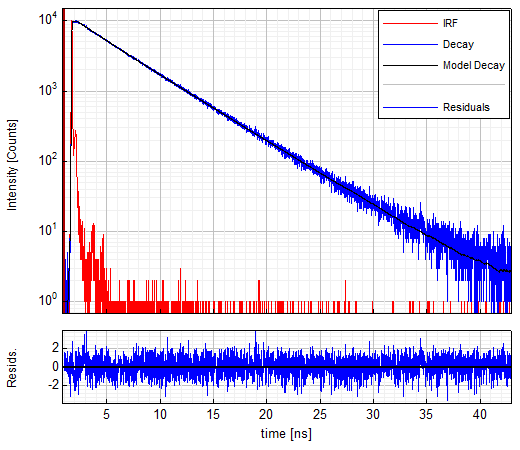

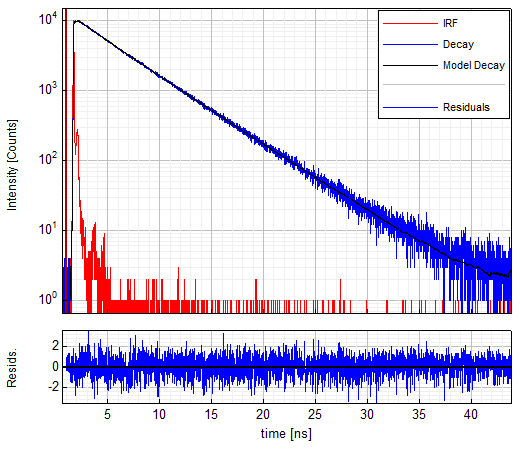


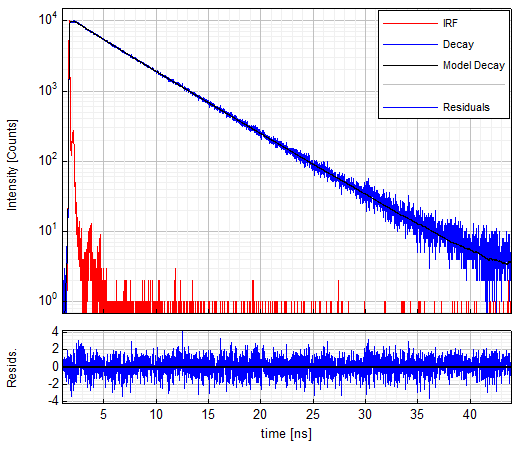

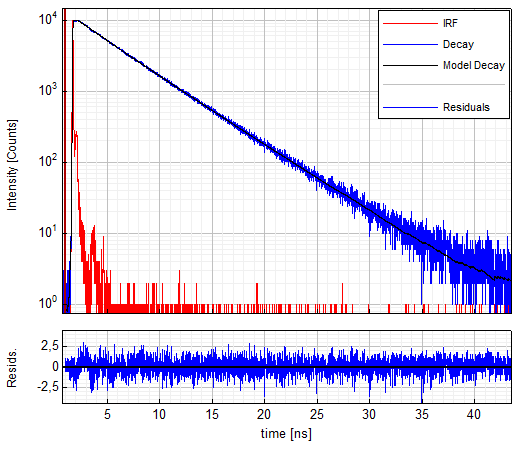


**dsCC**

**ατ**0.9154.870.0852.06

χ^2^(red) = 0.972

**dsGUUU**

**ατ**0.8734.500.1272.23

χ^2^(red) = 0.954

**dsGAUU**

**ατ**0.8854.620.1152.18

χ^2^(red) = 0.971

**dsUUUG**

**ατ**0.8904.340.1101.91

χ^2^(red) = 0.978

**dsAA**

**ατ**0.8884.600.1122.14

χ^2^(red) = 0.983

**dsAU**

**ατ**0.8994.420.1012.04

χ^2^(red) = 0.940


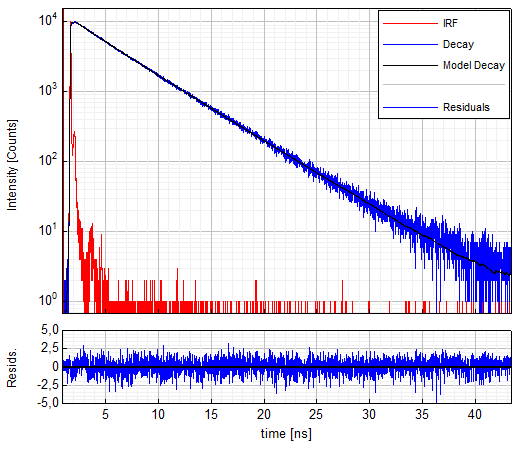

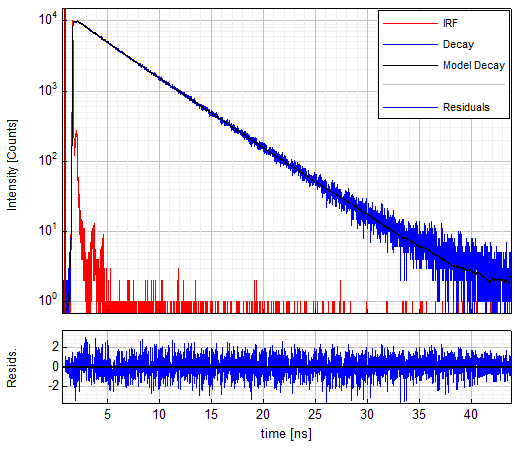


**Supplementary Figure S3. Fitted fluorescence decays of tC^O^ in double stranded RNA (cont.).**.

**Synthesis and characterization data**

**5-bromo-4-((2-hydroxyphenyl)amino)-1-(β-d-ribofuranosyl)pyrimidin-2(1*H*)-one (2):**

Dry MeCN (60 mL) and DIPEA (3.93 mL, 22.5 mmol, 3 equiv.) was added under inert conditions to a reaction vessel containing 2′,3′,5′-tri-*O*-acetyl-5-bromouridine (**1**, 3.37 g, 7.5 mmol) and 2-mesitylenesulfonyl chloride (2.30 g, 10.5 mmol, 1.4 equiv.), and the reaction was left to stir at room temperature for 4 hours. 2‑Aminophenol (1.64 g, 15.0 mmol, 2 equiv.) was added and the reaction was stirred for another 30 min, where after the mixture was concentrated and purified by column chromatography (silica gel, 0-20% EtOAc in DCM) to give a pale yellow solid, which was washed with 20% MeOH in DCM (2 × 10 mL) to give 2.49 g of the product (**2**) as a white solid. The filtrate was concentrated and purified by column chromatography (silica gel, 0-20% EtOAc in DCM) to give additional 380 mg of **2** as a white solid. Combined yield: 2.87 g (71%); ^1^H NMR (500 MHz, DMSO-*d*_6_): 10.20 (s, 1H), 8.48 (s, 1H), 8.24 (s, 1H), 8.06 (dd, *J* = 8.1, 1.6 Hz, 1H), 7.04 (td, *J* = 7.7, 1.7 Hz, 1H), 6.93 (dd, *J* = 8.1, 1.4 Hz, 1H), 6.85 (td, *J* = 7.7, 1.4 Hz, 1H), 5.90 (d, *J* = 4.5 Hz, 1H), 5.50 (dd, *J* = 6.3, 4.5 Hz, 1H), 5.37 (t, *J* = 6.1 Hz, 1H), 4.38 – 4.33 (m, 1H), 4.29 – 4.23 (m, 2H), 2.07 (s, 3H), 2.07 (s, 3H), 2.06 (s, 3H); ^13^C NMR (126 MHz, DMSO-*d*_6_): δ 170.0, 169.33, 169.30, 157.5, 152.9, 148.6, 143.0, 125.63, 125.55, 122.9, 119.0, 115.1, 89.6, 88.5, 79.1, 72.4, 69.6, 63.0, 20.6, 20.30, 20.27; HRMS (FAB+) calcd. for [C_21_H_22_BrN_3_NaO_9_]: 562.0437, found: 562.04.

**3-(β-d-ribofuranosyl)-3*H*-benzo[*b*]pyrimido[4,5-*e*][1,4]oxazin-2(10*H*)-one (3):**

Ethanol (99.7%, 23 mL) was added to a 20 mL microwave reaction vial containing 2 (2.82 g, 5.22 mmol) and KF (0.91 g, 3 equiv.). The vial was sealed with a cap and heated to 140 °C for 2 hours in a microwave reactor (Biotage® Initiator). The mixture was filtered and the solids washed with water (2 × 20 mL), and EtOH (2 × 10 mL) to afford 1.13 g of the product (3) as a tan solid after drying. The combined supernatant and washings were concentrated, and the resulting residue was suspended in water (20 mL) and filtered. The solids were washed with water (2 × 5 mL) and EtOH (2 × 5 mL), to afford additional 370 mg of 3 as a tan solid. Combined yield: 1.50 g (86%); ^1^H NMR (400 MHz, DMSO-*d*_6_): δ 10.64 (s, 1H), 7.63 (s, 1H), 6.91 – 6.74 (m, 4H), 5.75 (d, *J* = 3.6 Hz, 1H), 5.33 (d, *J* = 4.5 Hz, 1H), 5.20 (t, *J* = 4.8 Hz, 1H), 5.03 (d, *J* = 4.2 Hz, 1H), 4.01 – 3.92 (m, 2H), 3.86 – 3.79 (m, 1H), 3.70 – 3.62 (m, 1H), 3.60 – 3.53 ppm (m, 1H); ^13^C NMR (100.6 MHz, DMSO-*d*_6_): δ 153.2, 152.8, 142.3, 127.9, 127.0, 123.9, 123.6, 121.2, 117.4, 115.0, 88.7, 84.3, 73.9, 69.5, 60.5; HRMS (FAB+) calcd. for [C_15_H_15_N_3_NaO_6_]: 356.0859, found: 356.09.

**3-(5′-*O*-(4,4'-dimethoxytrityl)-β-d-ribofuranosyl)-3*H*-benzo[*b*]pyrimido[4,5-*e*][1,4]oxazin-2(10*H*)-one (3a):**

Dry pyridine (15.6 mL) was added to a reaction vessel containing compound 3 (0.78 g, 2.34 mmol) that was sealed with a septum and placed in an ice bath. DMT-Cl (952 mg, 2.81 mmol, 1.2 equiv.) was added in one portion, and the vessel was allowed to return to room temperature. After 1.5 h, the reaction mixture was partitioned between EtOAc (50 mL) and satd. aq. NaHCO_3_ (50 mL). The organic phase was washed with satd. aq. NaHCO_3_ (50 mL) and brine (50 mL) and evaporated in vacuo. The residue was dissolved in DCM and purified by column chromatography (silica gel, 2% MeOH in DCM) to give 1.07 g (72%) of the product (**3a**) as a pale yellow foam; ^1^H NMR (400 MHz, THF-*d*_8_): δ 10.29 (br s, 1H), 7.48 – 7.44 (m, 2H), 7.34 (dd, *J* = 8.8, 3.7 Hz, 4H), 7.28 (s, 1H), 7.22 (t, *J* = 7.7 Hz, 2H), 7.16 – 7.09 (m, 2H), 6.83 – 6.68 (m, 7H), 6.44 – 6.37 (m, 1H), 5.72 (d, *J* = 3.2 Hz, 1H), 5.26 – 5.15 (br m, 1H), 4.25 – 4.18 (m, 2H), 4.14 – 4.09 (m, 1H), 4.04 – 3.99 (m, 1H), 3.68 (s, 3H), 3.66 (s, 3H), 3.40 (dd, *J* = 10.6, 4.0 Hz, 1H), 3.30 (dd, *J* = 10.6, 2.3 Hz, 1H); ^13^C NMR (100.6 MHz, THF-*d*_8_): δ 159.80, 159.76, 155.2, 154.3, 146.0, 143.9, 137.1, 136.7, 131.1, 131.0, 129.2, 129.0, 128.8, 128.6, 127.5, 124.7, 124.1, 121.7, 119.6, 115.8, 114.0, 113.9, 91.7, 87.6, 84.3, 76.2, 71.1, 63.8, 55.42, 55.39; HRMS (FAB+) calcd. for [C_36_H_33_N_3_NaO_8_]: 658.2165, found: 658.22.

**3-(2′-*O*-(*tert*-butyldimethylsilyl)-5′-*O*-(4,4′-dimethoxytrityl)-β-d-ribofuranosyl)-3*H*-benzo[*b*]pyrimido[4,5-*e*][1,4]oxazin-2(10*H*)-one (3b):**

Compound 3a (1.10 mmol, 700 mg) and AgNO_3_ (224 mg, 1.32 mmol, 1.2 equiv.) were dissolved in dry THF (4 mL) and dry pyridine (0.4 mL). *tert*-Butyldimethylsilylchloride (199 mg, 1.32 mmol, 1.2 equiv.) was added in one portion and the mixture was left to react for 4 hours at room temperature. The reaction was quenched by MeOH, filtered and washed with EtOAc (2 × 5 mL). The filtrate was concentrated in vacuo, and the residue was purified by column chromatography (silica gel, EtOAc in heptane: 20-60%) to yield 627 mg (76%) of the product (**3b**) as a white solid; ^1^H NMR (400 MHz, CDCl_3_): δ 7.61 (dd, *J* = 7.9, 1.6 Hz, 1H), 7.50 (s, 1H), 7.46 – 7.43 (m, 2H), 7.37 – 7.29 (m, 6H), 7.26 – 7.21 (m, 1H), 6.89 – 6.83 (m, 5H), 6.79 (td, *J* = 7.8, 1.6 Hz, 1H), 6.42 (dd, *J* = 8.0, 1.4 Hz, 1H), 5.97 (d, *J* = 3.9 Hz, 1H), 4.36 (t, *J* = 5.1 Hz, 1H), 4.24 – 4.18 (m, 1H), 4.12 – 4.08 (m, 1H), 3.76 (s, 3H), 3.75 (s, 3H), 3.52 (dd, *J* = 11.0, 2.5 Hz, 1H), 3.30 (dd, *J* = 10.9, 3.1 Hz, 1H), 3.16 – 3.08 (m, 1H), 0.85 (s, 9H), 0.08 (s, 3H), -0.02 (s, 3H); ^13^C NMR (100.6 MHz, CDCl_3_): δ 158.71, 158.67, 155.4, 154.0, 144.5, 142.5, 135.9, 135.6, 130.2, 130.1, 128.3, 128.13, 128.10, 127.1, 126.4, 124.2, 123.7, 121.8, 118.4, 115.3, 113.44, 113.40, 90.0, 87.1, 83.4, 76.7, 70.2, 62.2, 55.29, 55.26, 26.0, 18.3, -4.3, -5.2; HRMS (FAB+) calcd. for [C_42_H_47_N_3_NaO_8_Si]: 772.3030, found: 772.30.

**3-(2′-*O*-(*tert*-butyldimethylsilyl)-3′-*O*-[(2-cyanoethyl-*N*,*N*-diisopropyl)phosphoramidyl]-5′-*O*-(4,4′-dimethoxytrityl)-β-d-ribofuranosyl)-3*H*-benzo[*b*]pyrimido[4,5-*e*][1,4]oxazin-2(10*H*)-one (4):**

Compound **3b** (0.23 mmol, 177 mg), 2-cyanoethyl *N*,*N*-diisopropylchlorophosphoramidite (126 µL, 0.45 mmol, 2.0 equiv.) and DIPEA (176 µL, 1.01 mmol, 4.5 equiv.) were dissolved in THF (5 mL) under argon. The reaction was left for 3 h where after distilled DCM (20 ml) was added. The mixture was washed with degassed saturated aqueous KCl, dried over MgSO_4_ and evaporated in vacuo. The crude mixture was purified under inert conditions by column chromatography (silica, EtOAc in hexane: 20% with 0.5% pyridine) to receive the two diastereomers (**4**) in 85% yield; ^31^P NMR (CDCl_3_): δ 149.7, 149.3.

### Spectral Data

**5-bromo-4-((2-hydroxyphenyl)amino)-1-(β-d-ribofuranosyl)pyrimidin-2(1*H*)-one (2):**


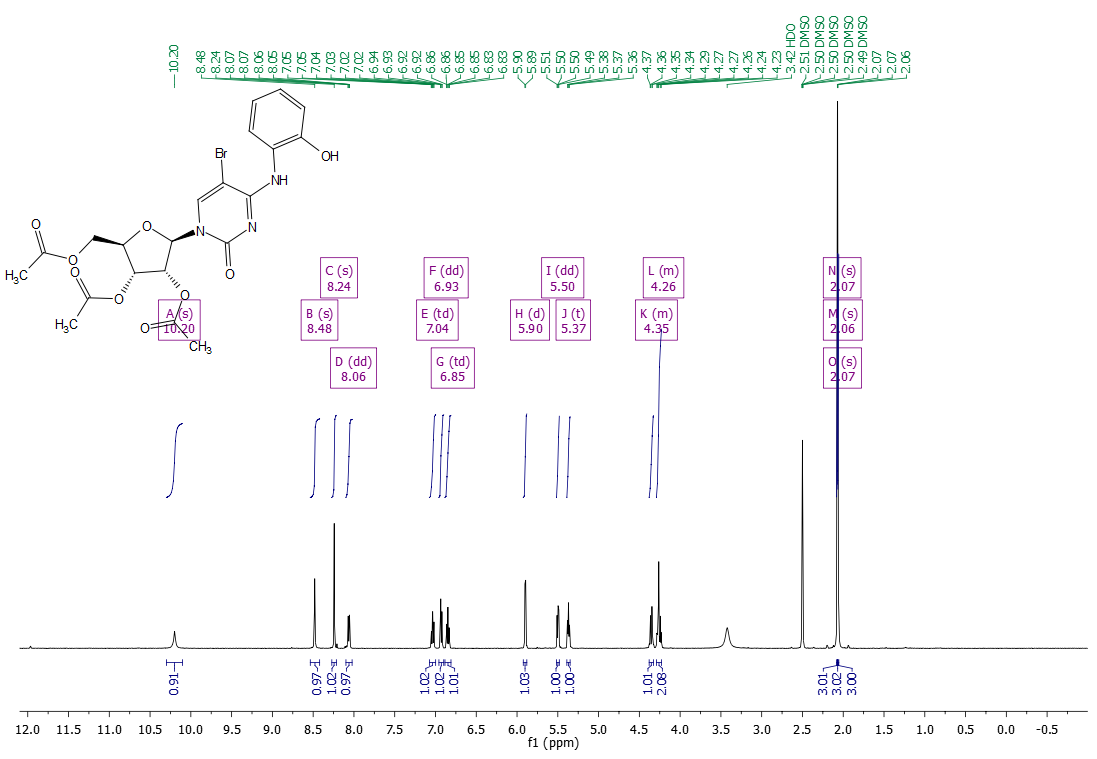


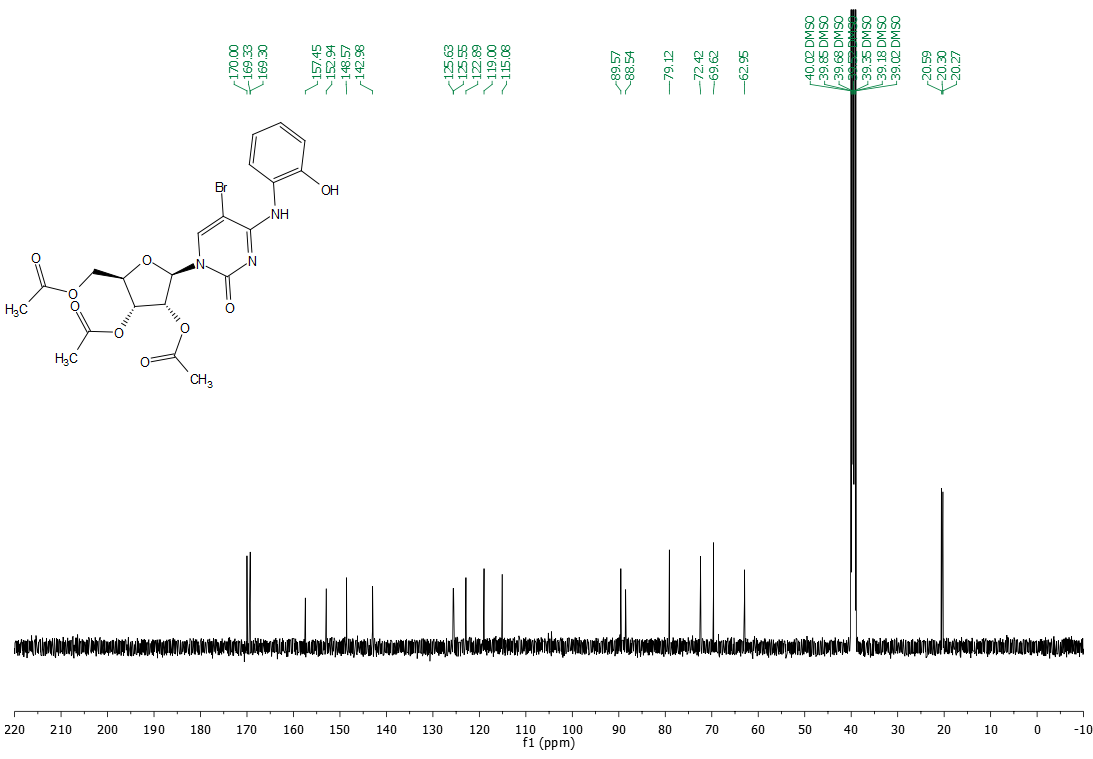


**3-(β-d-ribofuranosyl)-3*H*-benzo[*b*]pyrimido[4,5-*e*][1,4]oxazin-2(10*H*)-one (3):**

###
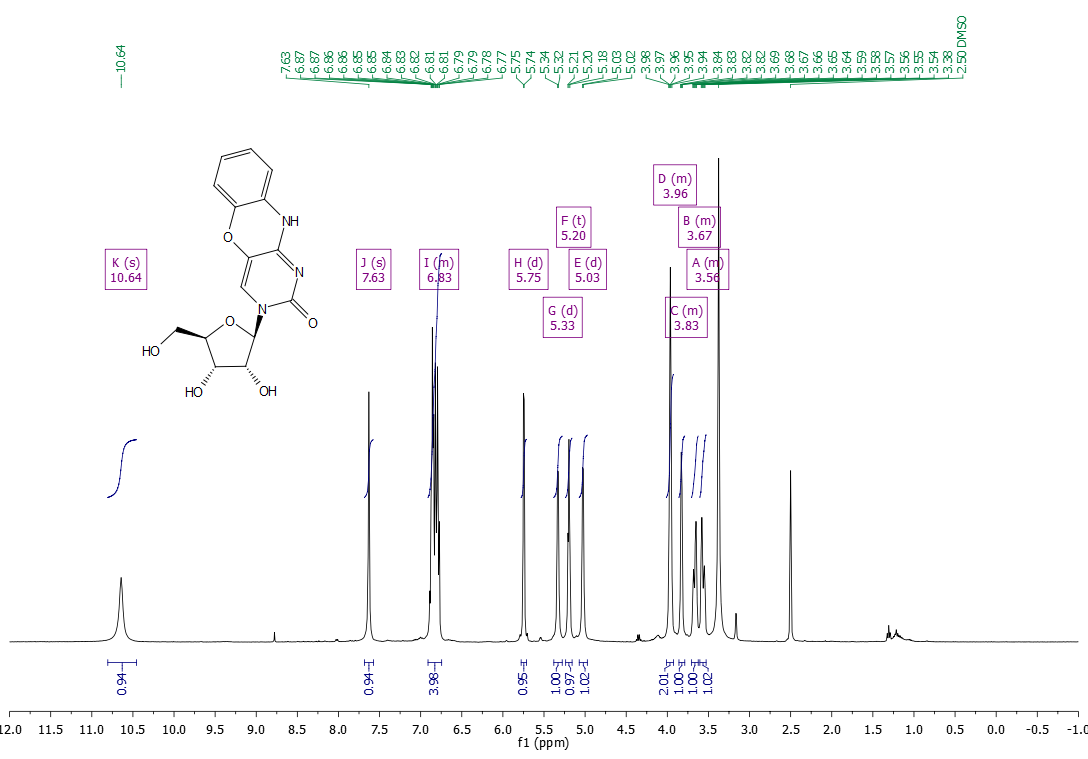

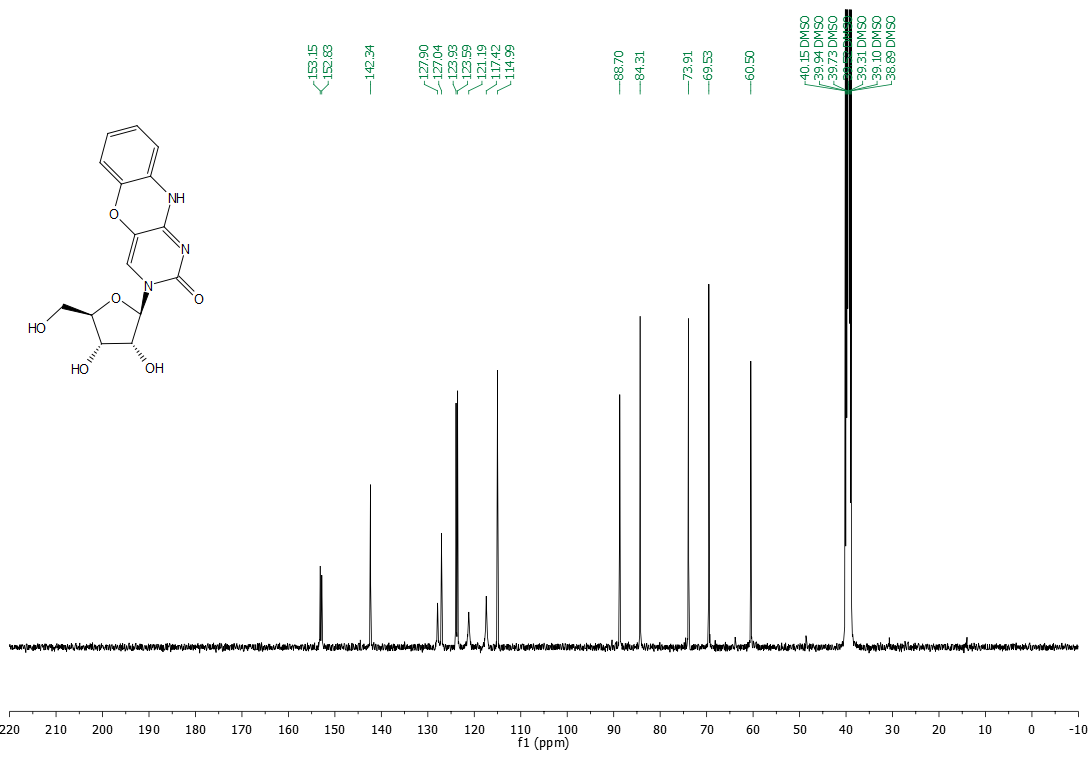


**3-(5′-*O*-(4,4'-dimethoxytrityl)-β-d-ribofuranosyl)-3*H*-benzo[*b*]pyrimido[4,5-*e*][1,4]oxazin-2(10*H*)-one (3a):**


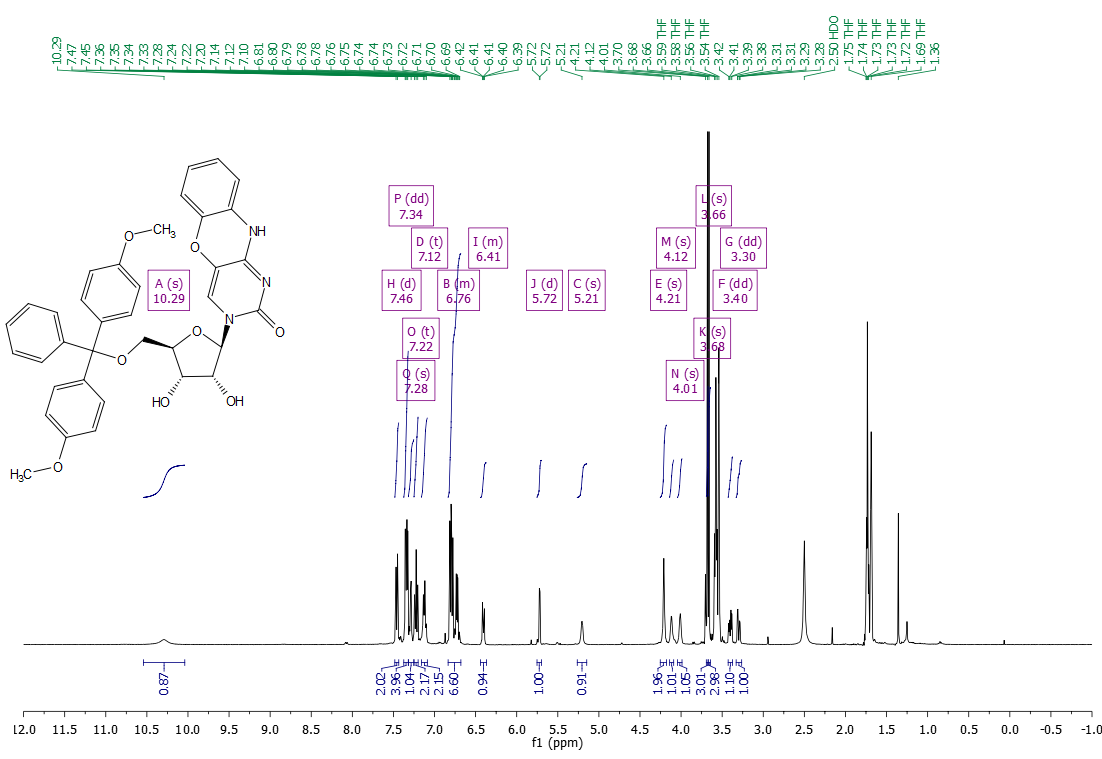

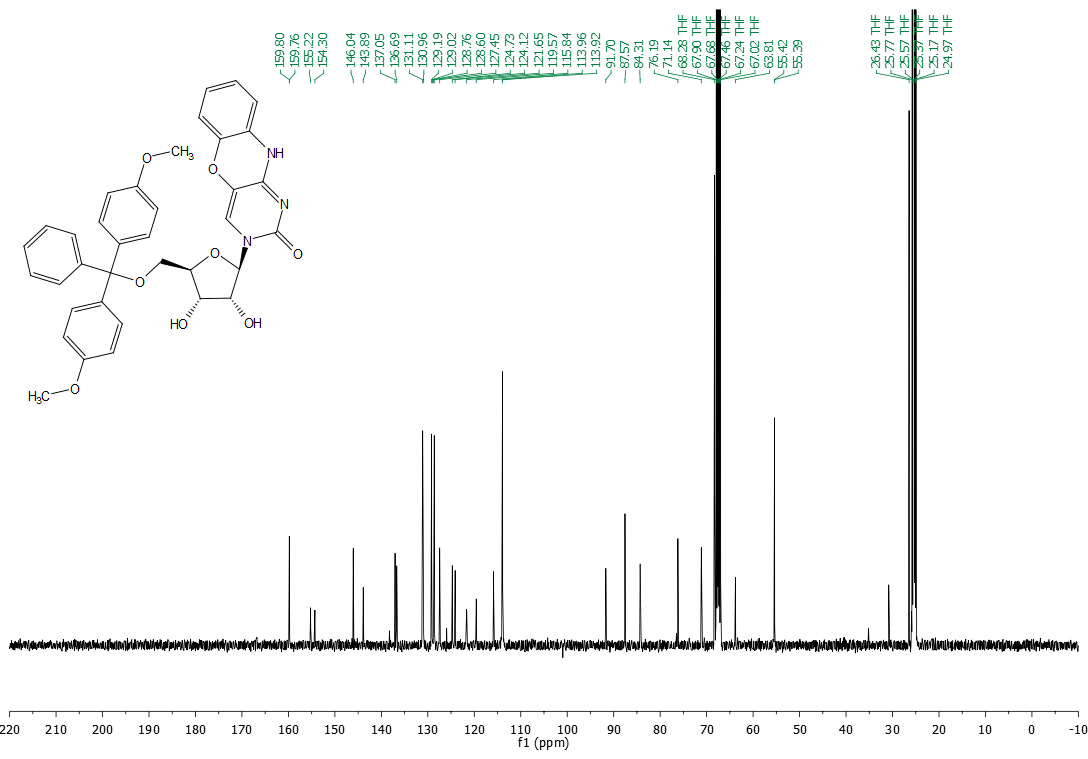


**3-(2′-*O*-(*tert*-butyldimethylsilyl)-5′-*O*-(4,4′-dimethoxytrityl)-β-d-ribofuranosyl)-3*H*-benzo[*b*]pyrimido[4,5-*e*][1,4]oxazin-2(10*H*)-one (3b):**


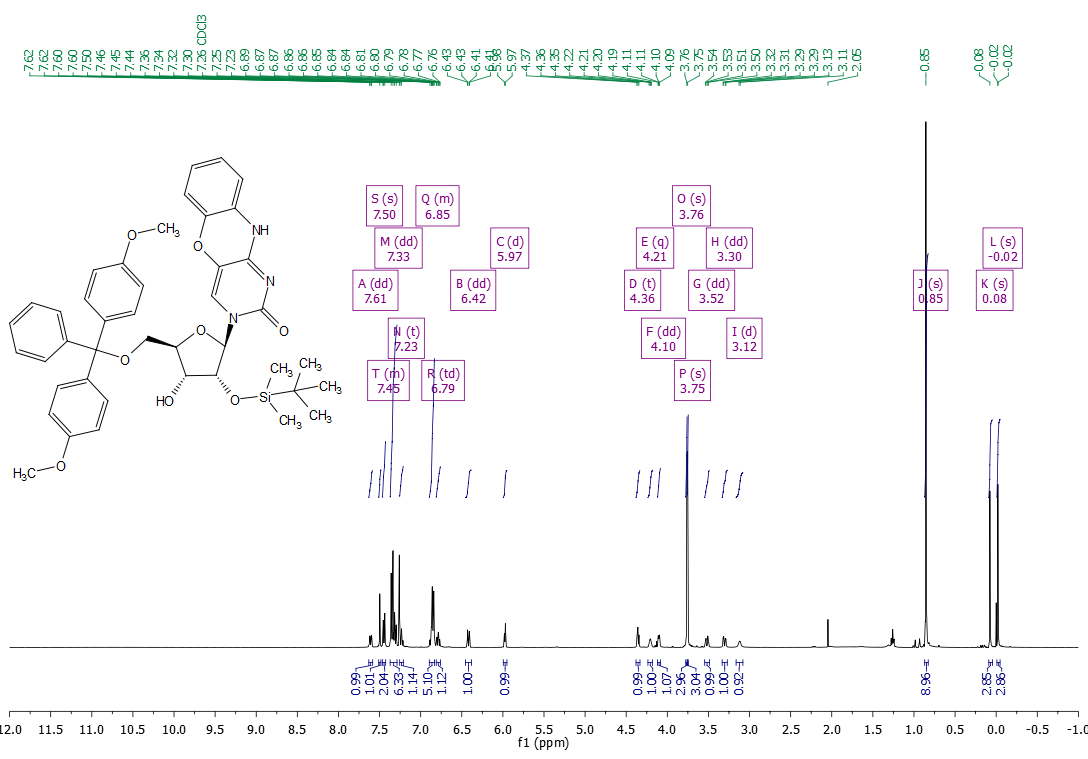

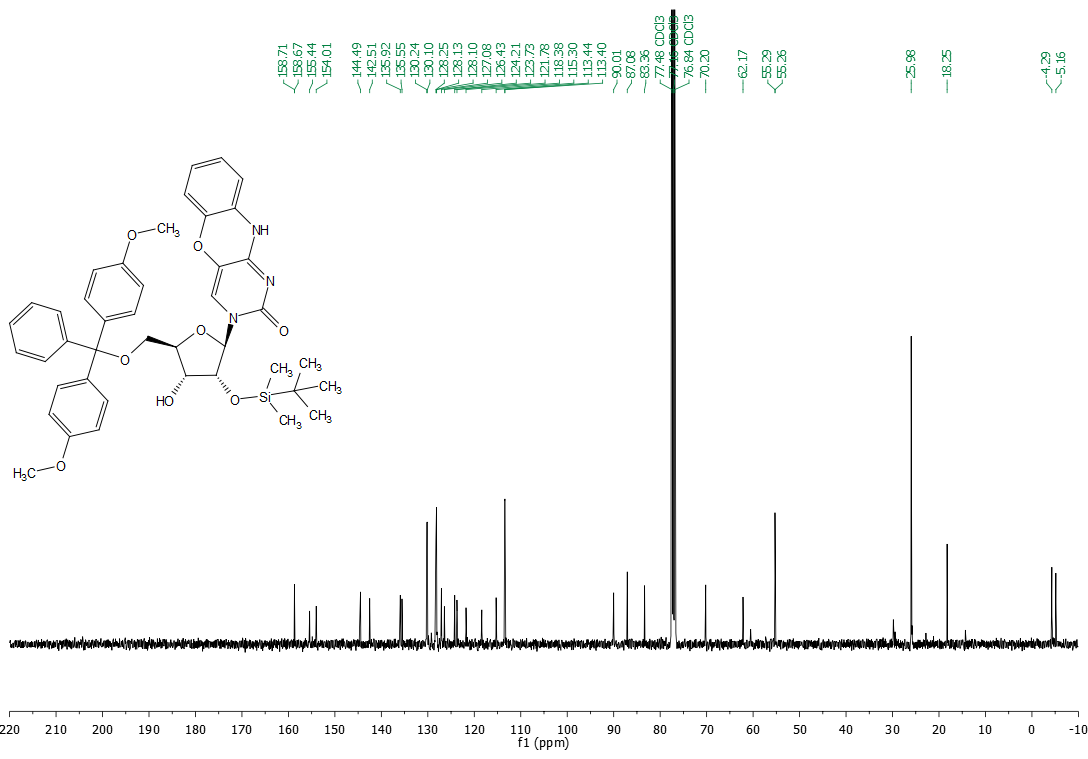


**3-(2′-*O*-(*tert*-butyldimethylsilyl)-3′-*O*-[(2-cyanoethyl-*N*,*N*-diisopropyl)phosphoramidyl]-5′-*O*-(4,4′-dimethoxytrityl)-β-d-ribofuranosyl)-3*H*-benzo[*b*]pyrimido[4,5-*e*][1,4]oxazin-2(10*H*)-one (4):**

###
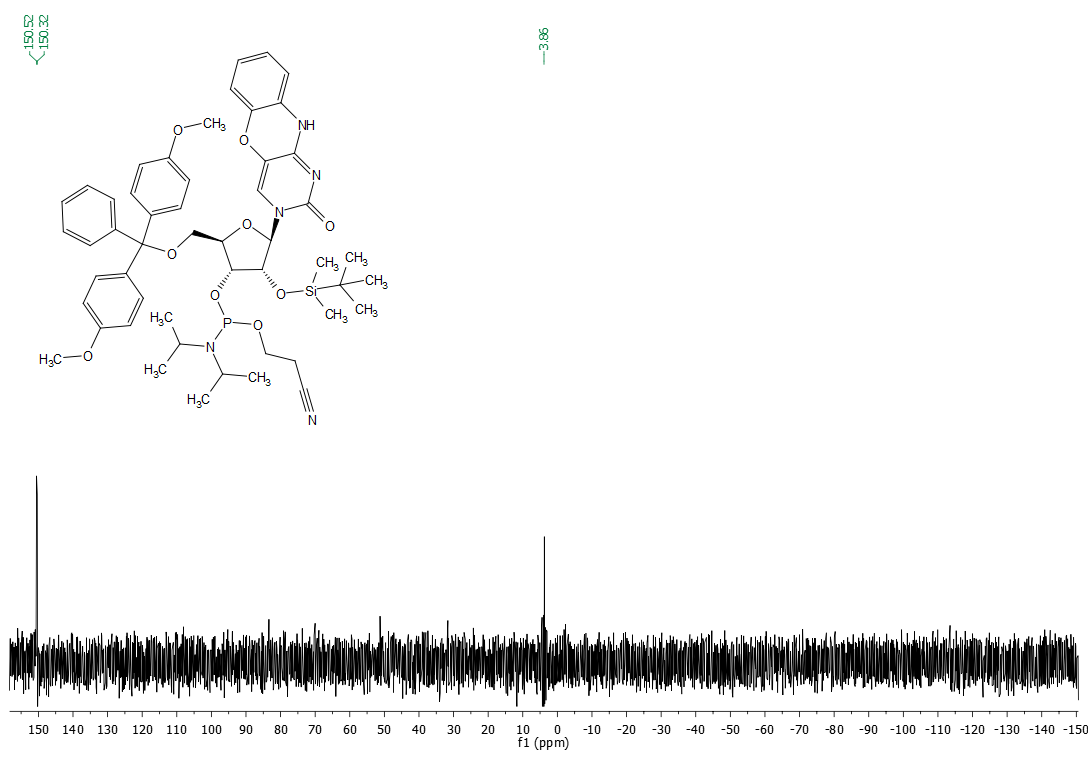


### References

1. Beaucage, S.L. and Caruthers, M.H. (1981) Deoxynucleoside phosphoramidites-a new class of key intermediates for deoxypolynucleotide synthesis. *Tetrahedron Lett.*, **22**, 1859–1862.
